# Exploratory Case Study of Suicide among a Sample of 9/11 Survivors

**DOI:** 10.3390/ijerph19010057

**Published:** 2021-12-22

**Authors:** Kacie Seil, Erin Takemoto, Mark R. Farfel, Mary Huynh, Jiehui Li

**Affiliations:** 1 New York City Department of Health & Mental Hygiene, World Trade Center Health Registry, Queens, NY 11101, USA; coburn.ee@gmail.com (E.T.); mfarfel@health.nyc.gov (M.R.F.); jli3@health.nyc.gov (J.L.); 2 New York City Department of Health & Mental Hygiene, Bureau of Vital Statistics, New York, NY 10013, USA; mhuynh@health.nyc.gov

**Keywords:** suicide, 9/11 disaster, World Trade Center, mortality, case study, data linkage

## Abstract

Background: Previous research has found higher than expected suicide mortality among rescue/recovery workers (RRWs) enrolled in the World Trade Center Health Registry (WTCHR). Whether any enrollee suicides are related to the decedents’ experiences on 9/11 is unknown. We abstracted medical examiner file data to learn more about 9/11-related circumstances of suicides among WTCHR enrollees. Methods: We identified 35 enrollee suicide cases that occurred in New York City using linked vital records data. We reviewed medical examiner files on each case, abstracting demographic and circumstantial data. We also reviewed survey data collected from each case at WTCHR enrollment (2003–2004) and available subsequent surveys to calculate descriptive statistics. Results: Cases were mostly non-Hispanic White (66%), male (83%), and middle-aged (median 58 years). Nineteen decedents (54%) were RRWs, and 32% of them worked at the WTC site for >90 days compared to 18% of the RRW group overall. In the medical examiner files of two cases, accounts from family mentioned 9/11-related circumstances, unprompted. All deaths occurred during 2004–2018, ranging from one to four cases per year. Leading mechanisms were hanging/suffocation (26%), firearm (23%), and jump from height (23%). Sixty percent of the cases had depression mentioned in the files, but none mentioned posttraumatic stress disorder. Conclusions: RRWs may be at particular risk for suicide, as those who worked at the WTC site for long periods appeared to be more likely to die by suicide than other RRWs. Mental health screening and treatment must continue to be prioritized for the 9/11-exposed population. More in-depth investigations of suicides can elucidate the ongoing impacts of 9/11.

## 1. Introduction

Suicide is a worsening public health crisis in the United States [1]. Studies on suicide trends in New York and New York City (NYC) shortly after the 11 September 2001 terrorist attacks have shown mixed results–mainly unchanging [2] or decreasing trends [3,4]. Longer-term suicide trends related to 9/11 are largely unknown, but even knowledge of trends does not provide an understanding of suicide circumstances occurring among 9/11 survivors.

Long-term studies on 9/11 survivors and disaster-exposed populations, generally, are important because established risk factors for suicide (posttraumatic stress disorder [PTSD] [5,6,7], depression [8], substance misuse [9,10,11]) are reported to be highly prevalent 10–15 years later, suggesting that this population may be differentially at risk over time. Research on World Trade Center Health Registry (WTCHR) enrollees has found that certain 9/11-related exposures were associated with greater odds of alcohol- and drug-related mortality among enrollees [12]. Previous research on WTCHR enrollees has also found higher than expected suicide standardized mortality ratios among rescue/recovery workers (RRWs) compared to the NYC population [13], and RRWs with PTSD had a 2.5-fold increase in risk of suicide versus those without PTSD [14]. More detailed analysis on suicide circumstances in the 9/11-exposed population has not been done.

This exploratory case study aimed to learn more about potential 9/11-related circumstances of suicides in a sample of WTCHR enrollees over a 14-to-15-year period using a linkage of multiple data sources including NYC Office of Chief Medical Examiner (OCME) files, vital records, and WTCHR survey data.

## 2. Methods

The WTCHR is a longitudinal cohort study established in 2002 that enrolled over 71,000 people exposed to the 9/11 disaster in 2003–2004 in NYC to better understand short- and long-term health effects. Using the most recently available NYC linked vital records data, we identified 35 WTCHR enrollee suicide cases (ICD-10 underlying cause of death codes: X60-X84, Y87.0, U03) out of 2545 enrollee deaths that occurred after enrollment, during 2004–2018 in NYC. We abstracted OCME file data, constructing a database that included some elements referenced in the National Violent Death Reporting System coding manual [15]. OCME case worksheets, investigation reports, death certificates, supplemental information forms, autopsy results, and toxicology results were reviewed for mention of circumstances related to 9/11 (e.g., being a RRW, losing a loved one, having 9/11-related PTSD). We also abstracted data on co-morbid physical or mental health conditions and other traumatic or stressful life events from OCME file data. We used WTCHR Wave 1 (baseline) survey data, which included enrollees’ self-reported health and 9/11-specific exposure information at the time of enrollment, and subsequent available wave data for their latest reports on health conditions before the event occurred. Mental health screening scales were also included in WTCHR surveys and completed by all respondents. Using data from these sources and vital records data, we calculated descriptive statistics (medians, frequencies) for the decedents’ demographic characteristics, 9/11-related exposures, health outcomes, and suicide circumstances.

Institutional Review Boards at the Centers for Disease Control and Prevention and the New York City Department of Health and Mental Hygiene (NYC DOHMH) approved the Registry protocol; NYC DOHMH approved this study.

## 3. Results

The 35 suicide cases were mostly non-Hispanic White (66%), male (83%), and middle-aged (median 58 years) (Table 1). The race/ethnicity, sex, and age patterns of enrollees who died by other causes differed in that they were much less likely to be non-Hispanic White (49%), male (55%), and middle-aged (median 68 years). Leading mechanisms of suicide were hanging/suffocation (26%), firearm (23%), and jump from height (23%). Over half (*N* = 19, 54%) of the enrollees who died by suicide were RRWs; the remaining were lower Manhattan residents (29%) and area workers or passers-by (17%) on the morning of 9/11. A much smaller proportion of enrollees who died of non-suicide causes were RRWs: 24%. Of the RRWs who died by suicide, 32% had worked at the WTC site for more than 90 days compared to 18% of the RRW group overall, although the difference was not statistically significant based on a two-sided *p*-value level of <0.05 (Table 2).

**Figure 1:**
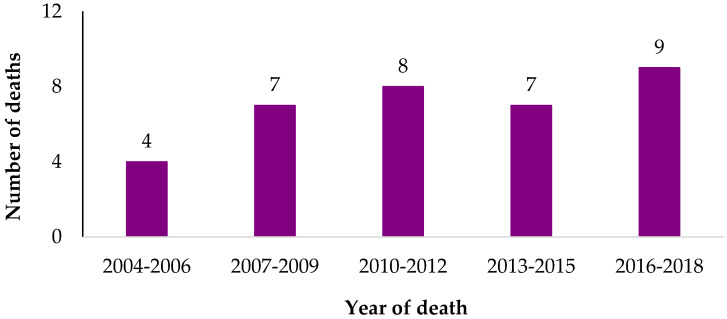
Number of WTCHR enrollee suicide deaths in NYC by year. WTCHR: World Trade Center Health Registry.

Suicide deaths occurred between 2004 and 2018, ranging from one to four cases per year (Figure 1). Supplemental information in the OCME files provided by family members mentioned relevant 9/11 circumstances for two cases. Neither were RRWs, and both had severe chronic physical health conditions according to the next of kin accounts. Sixty percent of suicide cases had depression mentioned in the OCME files, but none mentioned PTSD. Seven of the cases (20%) met criteria for probable 9/11-related PTSD based on a Post-Traumatic Stress Disorder Checklist (PCL-17) score of 44 or more in any of the four WTCHR survey waves [16], and six of them had probable PTSD at the last wave survey they participated in. Seven cases (20%) met the criteria for severe nonspecific psychological distress symptoms based on a Kessler Scale (K6) score of 13 or more [17].

When stratifying by eligibility group (data not shown), there were some differences in sociodemographic patterns, though the numbers are small. Females were 17% of the overall cases, but 67% (*N* = 4) of area workers and passers-by were females; area workers and passers-by were also more likely to be non-Hispanic White than the group overall (83% vs. 66%, respectively). Seven out of the eight suicides by firearm occurred in the RRW group. The most common Standard Occupational Classification (SOC) categories [18] among the cases based on OCME file information were: protective service (*N* = 5), business and financial operations (*N* = 4), office and administrative support (*N* = 4), transportation and material moving (*N* = 4), and construction and extraction (*N* = 3).

## 4. Discussion

This exploratory case study utilized multiple data sources to better understand characteristics and circumstances of suicide deaths among a sample of 9/11-exposed individuals. The OCME data revealed that experiences on 9/11 were potentially related to two suicides among WTCHR enrollees based on unprompted information about 9/11 circumstances reported by family members of each decedent. These deaths occurred more than ten years after 9/11, meaning that 9/11-related exposures and health conditions, particularly serious ones, may be relevant risk factors for suicide to this day. Notably, both individuals were not RRWs.

The overall demographic patterns were similar between enrollees who died by suicide and NYC residents who died by suicide [19]. The proportion of firearm suicides may have been higher in our sample, however [19]; this may be related to the likelihood of having access to firearms in RRW/law enforcement types of occupations. The results also point to RRWs as a group that may be at increased risk for suicide, as the proportion of suicide cases that are RRWs is twice that of non-suicide deaths (54% vs. 24%, respectively). A systematic review examining suicidal thoughts and behaviors among first responders (i.e., police officers, emergency medical technicians, paramedics, and firefighters) concluded that the group was likely at greater risk than the general public, though there was a dearth of methodologically rigorous studies on the topic [20]. Other research has shown that a prolonged period of work at the WTC site was associated with greater risk of PTSD among NYC firefighters [5], so the effects of this exposure may be diverse and long-lasting. Our results demonstrated a pattern of longer periods of work at the WTC site among RRWs who died by suicide, but the numbers did not reach a level of statistical significance.

The Durkheim theory posits that suicide rates often decrease following large-scale disasters or traumatic events, as those affected may experience social cohesion [2]. This has been noted in many types of natural and man-made disasters [21,22,23,24], though some experts caution that those decreases can be followed by increases years later [25]. Recent research has shown that suicide rates began to increase in Japan in the latter half of 2020, likely due to impacts of the COVID-19 pandemic [26], though findings from many other countries have shown no increases thus far [27]. Long-term tracking is therefore necessary. We now know that experiences on 9/11 were significant enough to be mentioned by family members in at least two enrollee suicide events more than a decade later. The disaster’s impact across the life-course can be substantial, particularly for those suffering from serious mental or physical health conditions.

Use of multiple data sources helped us to better understand the circumstances of suicide cases over the years among NYC enrollees. This sample was limited to WTCHR enrollees who died in NYC sometime after enrollment, so it is a subset of all 9/11-exposed individuals who died by suicide. The level of detail on suicide circumstances in the OCME files also differed by case. Although history of depression was reported in the majority of decedents’ medical examiner files, there was rarely information on whether it began after 9/11, how long the condition lasted, or whether there was a family history. Some files contained information on decedents’ previous mental health treatments, but since the data were reported with great variation, we did not include that information in this study. In this study, the number of suicides was too small to allow for calculation of age-adjusted rates. The small sample size also limited our ability to make comparisons between RRWs who died by suicide vs. all other RRWs, though we feel that the results in Table 2 indicate a pattern worth examining more closely.

## 5. Conclusions

Suicide is an ongoing issue for 9/11 survivors, with experiences and health impacts related to the unprecedented disaster possibly playing a role in some deaths. RRWs may be at particular risk, as those who worked at the WTC site for long periods appeared to be more likely to die by suicide than other RRWs. This risk factor should be further investigated in a larger sample with more years of data. A history of depression among the 35 cases was common, warranting continued prioritization of mental health screenings and treatment for this population. Although a history of PTSD was not reported in any of the decedents’ medical examiner files, our survey data show that at least seven of them (20%) had probable PTSD at some point prior to death.

Future studies could be conducted on enrollees who died outside of NYC to increase the validity of our findings. Additionally, a future qualitative study on all suicide deaths in NYC (not just WTCHR enrollees) could help us examine whether 9/11 was a circumstance in any other suicides. Enrollees’ experiences during the COVID-19 pandemic might also be relevant to existing or future suicide risk. A study of self-inflicted injuries among WTCHR enrollees is currently underway, which could help identify those in need of suicide prevention resources. Future research will allow us to continue elucidating the ongoing impact of 9/11 and opportunities for intervention.

## Figures and Tables

**Table 1:** Demographic characteristics and potential 9/11-related mental health conditions among WTCHR enrollees who died by suicide vs. non-suicide in NYC during 2004–2018.

	Suicide Deaths (*N* = 35)		Non-Suicide Deaths (*N* = 2510)	
	*N*	%	*N*	%
Age at death in years, median (IQR)	58 (17)		68 (24)	
Sex				
Male	29	83%	1391	55%
Female	6	17%	1118	45%
Race/ethnicity				
Non-Hispanic White	23	66%	1226	49%
Non-Hispanic Black	5	14%	445	18%
Hispanic	4	11%	358	14%
Asian	3	9%	338	13%
Multiple/other	0	0%	142	6%
Eligibility group				
RRWs	19	54%	598	24%
Lower Manhattan residents	10	29%	1098	44%
Area workers & passers-by	6	17%	813	32%
Injury mechanism				
Hanging/suffocation	9	26%	-	-
Firearm	8	23%	-	-
Fall	8	23%	-	-
Poisoning	7	20%	-	-
Other	3	9%	-	-
History of reported depression °	21	60%	-	-
Mention of 9/11 by next of kin °	2	6%	-	-
Any self-report of probable PTSD (PCL-17 ≥ 44) ^†^	7	20%	818	33%
Probable PTSD at Wave 1	4	11%	614	24%
Any self-report of probable NSPD (K6 ≥ 13) ^†^	7	20%	534	21%
Probable NSPD at Wave 1	5	14%	389	15%

Note: sums may not equal 100% due to missing data. WTCHR: World Trade Center Health Registry; IQR: interquartile range; PTSD: posttraumatic stress disorder; NSPD: non-specific psychological distress; OCME: Office of Chief Medical Examiner; RRW: rescue/recovery worker. ° Data collected from OCME files. ^†^ Data collected from WTCHR wave surveys.

**Table 2:** 9/11 exposure characteristics of WTCHR RRWs who died by suicide in NYC in 2004–2018 vs. other WTCHR RRWs.

	WTCHR RRWs Who Died by Suicide		Other WTCHR RRWs		Fisher’s Exact Test *p*-Value
	*N*	%	*N*	%	
Days worked at WTC site †					
1–7 days	4	21%	10,201	36%	0.268
7.5–30 days	7	37%	8495	30%	
30.5–90 days	2	11%	4733	17%	
90.5–210.5 days	6	32%	5010	18%	

WTCHR: World Trade Center Health Registry; RRW: rescue/recovery worker. † Data collected from Wave 1 survey.

## Data Availability

World Trade Center Health Registry data may be made available following review of applications to the Registry from external researchers. Data from NYC DOHMH Bureau of Vital Statistics (vital records) and NYC Office of Chief Medical Examiner (OCME files) may be requested from those entities separately.

## References

[1] Stone D.M., Simon T.R., Fowler K.A., Kegler S.R., Yuan K., Holland K.M., Ivey-Stephenson A.J., Crosby A.E. (2018). Vital Signs: Trends in State Suicide Rates—United States, 1999–2016 and Circumstances Contributing to Suicide—27 States, 2015. MMWR Morb. Mortal. Wkly. Rep..

[2] Pridemore W.A., Trahan A., Chamlin M.B. (2009). No evidence of suicide increase following terrorist attacks in the United States: An interrupted time-series analysis of September 11 and Oklahoma City. Suicide Life Threat. Behav..

[3] Mezuk B., Larkin G.L., Prescott M.R., Tracy M., Vlahov D., Tardiff K., Galea S. (2009). The influence of a major disaster on suicide risk in the population. J. Trauma. Stress..

[4] Claassen C.A., Carmody T., Stewart S.M., Bossarte R.M., Larkin G.L., Woodward W.A., Trivedi M.H. (2010). Effect of 11 September 2001 terrorist attacks in the USA on suicide in areas surrounding the crash sites. Br. J. Psychiatry.

[5] Berninger A., Webber M.P., Cohen H., Gustave J., Lee R., Niles J.K., Chiu S., Zeig-Owens R., Soo J., Kelly K. (2010). Trends of elevated PTSD risk in firefighters exposed to the World Trade Center disaster: 2001–2005. Public Health Rep..

[6] Farfel M.R., Friedman S., Perlman S.E., Stellman S.D., Walker D.J., Wu D., Yu S., Thorpe L.E., Brackbill R.M., Ekenga C.C. (2009). Asthma and posttraumatic stress symptoms 5 to 6 years following exposure to the World Trade Center terrorist attack. Jama.

[7] Neria Y., DiGrande L., Adams B.G. (2011). Posttraumatic stress disorder following the September 11, 2001, terrorist attacks: A review of the literature among highly exposed populations. Am. Psychol..

[8] Caramanica K., Brackbill R.M., Liao T., Stellman S.D. (2014). Comorbidity of 9/11-related PTSD and depression in the World Trade Center Health Registry 10–11 years postdisaster. J. Trauma Stress..

[9] Dewart T., Frank B., Schmeidler J. (2006). The impact of 9/11 on patients in New York City’s substance abuse treatment programs. Am. J. Drug Alcohol Abuse..

[10] Vlahov D., Galea S., Ahern J., Resnick H., Boscarino J.A., Gold J., Bucuvalas M., Kilpatrick D. (2004). Consumption of cigarettes, alcohol, and marijuana among New York City residents six months after the September 11 terrorist attacks. Am. J. Drug Alcohol Abuse.

[11] Gargano L.M., Welch A.E., Stellman S.D. (2017). Substance use in adolescents 10 years after the World Trade Center attacks in New York City. J. Child Adolesc. Subst. Abuse.

[12] Welch A.E., Zweig K.C., Liao T., Yip J., Davidson A., Jordan H., Brackbill R., Cone J. (2018). Alcohol and Drug-Related Mortality Among Enrollees in the World Trade Center Health Registry (WTCHR), 2004 to 2012. J. Occup. Environ. Med..

[13] Jordan H.T., Stein C.R., Li J., Cone J.E., Stayner L., Hadler J.L., Brackbill R.M., Farfel M.R. (2018). Mortality among rescue and recovery workers and community members exposed to the September 11, 2001 World Trade Center terrorist attacks, 2003–2014. Environ. Res..

[14] Giesinger I., Li J., Takemoto E., Cone J.E., Farfel M.R., Brackbill R.M. (2020). Association Between Posttraumatic Stress Disorder and Mortality Among Responders and Civilians Following the September 11, 2001, Disaster. JAMA Netw. Open.

[15] Prevention CfDCa (2021). National Violent Death Reporting System (NVDRS) Coding Manual Revised. National Center for Injury Prevention and Control CfDCaP.

[16] Blanchard E.B., Jones-Alexander J., Buckley T.C., Forneris C.A. (1996). Psychometric properties of the PTSD Checklist (PCL). Behav. Res. Ther..

[17] Kessler R.C., Andrews G., Colpe L.J., Hiripi E., Mroczek D.K., Normand S.-L., Walters E.E., Zaslavsky A.M. (2002). Short screening scales to monitor population prevalences and trends in non-specific psychological distress. Psychol. Med..

[18] Statistics USBoL Occupational Employment and Wage Statistics: May 2020 Occupation Profiles. https://www.bls.gov/oes/current/oes_stru.htm#00-0000.

[19] Protacio A., Norman C. (2016). Suicides in New York City, 2000 to 2014. Hygiene NYCDoHaM.

[20] Stanley I.H., Hom M.A., Joiner T.E. (2016). A systematic review of suicidal thoughts and behaviors among police officers, firefighters, EMTs, and paramedics. Clin. Psychol. Rev..

[21] Gordon K.H., Bresin K., Dombeck J., Routledge C., Wonderlich J.A. (2011). The impact of the 2009 Red River Flood on interpersonal risk factors for suicide. Crisis.

[22] Orui M., Harada S., Hayashi M. (2014). Changes in suicide rates in disaster-stricken areas following the Great East Japan Earthquake and their effect on economic factors: An ecological study. Environ. Health Prev. Med..

[23] Salib E., Cortina-Borja M. (2009). Effect of 7 July 2005 terrorist attacks in London on suicide in England and Wales. Br. J. Psychiatry.

[24] Hierholzer E., Bellamy N., Mannix B. (2015). Traumatic stress and suicide after disasters. Center SDTA.

[25] Matsubayashi T., Sawada Y., Ueda M. (2013). Natural disasters and suicide: Evidence from Japan. Soc. Sci. Med..

[26] Sakamoto H., Ishikane M., Ghaznavi C., Ueda P. (2021). Assessment of Suicide in Japan During the COVID-19 Pandemic vs. Previous Years. JAMA Netw. Open.

[27] Pirkis J., John A., Shin S., DelPozo-Banos M., Arya V., Analuisa-Aguilar P., Appleby L., Arensman E., Bantjes J., Baran A. (2021). Suicide trends in the early months of the COVID-19 pandemic: An interrupted time-series analysis of preliminary data from 21 countries. Lancet Psychiatry.

